# Nutrient metabolic heterogeneity across renal stages in patients with type 2 diabetes mellitus and diabetic kidney disease

**DOI:** 10.3389/fnut.2026.1885766

**Published:** 2026-07-15

**Authors:** Jiaji Hang, Like Xu, Kerong Hu, Dongli Sun, Xiaohang Wang

**Affiliations:** Department of Endocrinology, The Affiliated Hospital of Yangzhou University, Yangzhou University, Yangzhou, China

**Keywords:** diabetic kidney disease, nutrient metabolism, precision nutrition, TG/HDL-C ratio, time in range, type 2 diabetes mellitus

## Abstract

**Objective:**

Diabetic kidney disease (DKD) is a nutrition-related complication of type 2 diabetes mellitus (T2DM) characterized by complex disturbances in glucose, lipid, purine, and protein-related metabolism. This study aimed to investigate nutrient metabolic heterogeneity across renal stages in patients with T2DM and DKD, with particular focus on time in range (TIR), serum uric acid (SUA), TG/HDL-C ratio, and serum albumin (ALB).

**Methods:**

This single-center cross-sectional study included 589 hospitalized patients with T2DM, including 128 patients without DKD and 461 patients with DKD. DKD patients were further stratified according to KDIGO eGFR categories. Continuous glucose monitoring-derived TIR, SUA, TG/HDL-C ratio, and ALB were evaluated as multidimensional nutrient metabolism indicators. Multivariable logistic regression analyses were performed to identify independent metabolic indicators associated with DKD. Renal-stage trend analyses and stratified analyses in G3 and G5 were further conducted to explore stage-specific metabolic patterns.

**Results:**

Compared with patients without DKD, patients with DKD were older, had longer diabetes duration, and showed greater use of glucose-lowering and lipid-lowering medications. Patients with DKD also showed paradoxically higher TIR levels. In the overall multivariable model, higher TIR was associated with lower odds of DKD (OR = 0.97, 95% CI: 0.95–0.99, *p* = 0.010), whereas higher SUA (OR = 1.21, 95% CI: 1.08–1.31, *p* < 0.001) and TG/HDL-C ratio (OR = 1.27, 95% CI: 1.08–1.40, *p* = 0.002) were associated with higher odds of DKD. Higher ALB was associated with lower odds of DKD (OR = 0.91, 95% CI: 0.87–0.96, *p* < 0.001). Across renal stages, TIR decreased from G1 to G4, whereas SUA and TG/HDL-C ratio increased progressively with worsening renal function. ALB showed the lowest level in G5. In stratified analyses, TIR, SUA, and TG/HDL-C ratio remained significantly associated with DKD in G3, while ALB was the only significant metabolic correlate in G5.

**Conclusion:**

DKD in T2DM is characterized by stage-specific nutrient metabolic heterogeneity involving glucose, purine, lipid, and protein-related nutritional metabolism. G3 may represent a renal stage characterized by more prominent glucose, uric acid, and lipid metabolic abnormalities, whereas G5 may be characterized by more evident nutritional deterioration. These findings should be interpreted as stage-specific associations rather than causal progression pathways because of the cross-sectional design.

**Clinical trial registration:**

https://www.chictr.org.cn/bin/home, identifier ChiCTR2500111406.

## 1 Introduction

Diabetes mellitus is a chronic metabolic disease characterized by hyperglycemia resulting from impaired insulin secretion and insulin resistance (1). With the increasing global burden of type 2 diabetes mellitus (T2DM), diabetes-related vascular complications have become a major public health challenge (2). Among these complications, diabetic kidney disease (DKD) is one of the most common and clinically important microvascular complications (3). DKD is also increasingly recognized as a nutrition-related complication characterized by complex disturbances in glucose, lipid, purine, and protein-related metabolism (4). However, the stage-specific heterogeneity of nutrient metabolism in DKD remains insufficiently understood.

DKD is a heterogeneous disease with diverse clinical phenotypes and progression patterns (5). Some patients present with predominant albuminuria, whereas others experience renal function decline with relatively mild albuminuria (6). This heterogeneity complicates risk assessment and individualized management. Although glycemic control remains a cornerstone of DKD prevention and treatment, traditional glycemic indicators such as glycated hemoglobin (HbA1c) have important limitations, particularly in patients with impaired renal function (7). Anemia, altered erythrocyte lifespan, blood transfusion, erythropoietin therapy, iron supplementation, and advanced kidney dysfunction may all affect HbA1c accuracy (8). Therefore, HbA1c alone may not fully capture glycemic exposure or glucose fluctuation in patients with DKD (9).

Continuous glucose monitoring (CGM) provides a more comprehensive assessment of glycemic patterns than HbA1c (10). Among CGM-derived metrics, time in range (TIR), defined as the proportion of time that glucose levels remain within the target range, has emerged as a clinically useful indicator of glycemic stability (11). Previous studies have reported associations between TIR and diabetic microvascular complications, including DKD (12). However, most existing studies have focused on the overall association between TIR and DKD, without sufficiently considering the heterogeneity introduced by renal function stage (13). This is clinically important because declining eGFR substantially alters glucose metabolism, insulin clearance, hypoglycemia susceptibility, treatment intensity, and the interpretation of glycemic metrics (14)^.^

In addition to glucose metabolism, DKD is closely related to broader nutrient metabolic disturbances. Uric acid metabolism, lipid metabolism, and nutritional status may all contribute to renal injury and systemic deterioration in T2DM (15). Serum uric acid (SUA) reflects purine metabolism and renal urate handling, while the triglyceride-to-high-density lipoprotein cholesterol ratio (TG/HDL-C) represents atherogenic dyslipidemia and insulin resistance-related lipid disturbance (16). Serum albumin (ALB), although influenced by multiple factors, is an important clinical marker related to nutritional status, inflammation, protein loss, and catabolic burden (17). Recent studies in Frontiers in Nutrition have increasingly emphasized the value of integrated metabolic indicators, such as remnant cholesterol and the triglyceride-glucose index, in evaluating diabetes-related complications from a nutritional metabolism perspective (18, 19). This approach suggests that DKD should not be interpreted solely as a glucose-related complication, but rather as a multidimensional metabolic disorder involving glucose, lipid, purine, and protein and nutritional pathways (20).

Renal function decline may further reshape the relative importance of these metabolic pathways. In earlier stages of renal impairment, glucose stability, uric acid burden, and lipid abnormalities may remain important metabolic targets (21). In advanced renal dysfunction, however, protein-energy wasting, inflammation, reduced appetite, altered glucose handling, and nutritional deterioration may become increasingly prominent (22). Current nutritional strategies for DKD commonly stratify protein intake according to renal function, but glycemic and lipid-related dietary recommendations are often applied in a relatively uniform manner (23). Whether the clinical relevance of TIR and other nutrient metabolism indicators differs across eGFR stages remains insufficiently explored (24). From a nutrition perspective, DKD progression may involve a transition from predominantly glucose and lipid-related metabolic disturbances toward protein-energy wasting and nutritional deterioration in advanced renal dysfunction (25).

Therefore, the present study aimed to investigate the association between TIR and DKD in patients with T2DM from a multidimensional nutrient metabolism perspective. Specifically, we evaluated the independent associations of TIR, SUA, TG/HDL-C ratio, and ALB with DKD, compared key metabolic indicators across renal stages, and further examined stage-specific metabolic patterns in G3 and G5. By doing so, this study sought to determine whether DKD is characterized by a stage-specific metabolic heterogeneity involving glucose, lipid, purine, and protein-related nutritional metabolism. Rather than evaluating TIR as an isolated glycemic marker, the present study aimed to characterize renal-stage-specific nutrient metabolic heterogeneity in DKD from a multidimensional metabolic perspective. Specifically, we evaluated glucose metabolism represented by TIR, purine metabolism represented by SUA, lipid metabolism represented by TG/HDL-C ratio, and nutrition-related status represented by ALB. We further explored whether these metabolic indicators showed different patterns between G3 and G5 renal stages.

## 2 Methods

### 2.1 Study design

This was a single-center cross-sectional study conducted at the Department of Endocrinology, the Affiliated Hospital of Yangzhou University. The study protocol was reviewed and approved by the Biomedical Ethics Review Committee of the Affiliated Hospital of Yangzhou University (Approval No. 2025-YKL07-K03).

### 2.2 Participants

A total of 589 patients with T2DM admitted to the Department of Endocrinology between December 2023 and January 2025 were included. T2DM was diagnosed according to the 1999 World Health Organization diagnostic criteria. DKD was diagnosed according to the 2021 Chinese Guidelines for the Prevention and Treatment of Diabetic Kidney Disease. Briefly, DKD was defined as diabetes accompanied by persistent albuminuria and/or reduced eGFR after exclusion of non-diabetic kidney diseases. Patients were divided into a non-DKD group (*n* = 128) and a DKD group (*n* = 461). Exclusion criteria were as follows: acute diabetic complications within the previous 3 months; pregnancy or lactation; severe infection; malignant tumors; acute heart failure; acute stroke; and incomplete clinical, CGM, or renal function data. Patients with DKD were further stratified according to KDIGO eGFR categories: G1 (eGFR ≥90 mL/min/1.73 m^2^, *n* = 125), G2 (60 ≤ eGFR<90 mL/min/1.73 m^2^, *n* = 140), G3a (45 ≤ eGFR<60 mL/min/1.73 m^2^, *n* = 55), G3b (30 ≤ eGFR<45 mL/min/1.73 m^2^, *n* = 50), G4 (15 ≤ eGFR<30 mL/min/1.73 m^2^, *n* = 51), and G5 (eGFR<15 mL/min/1.73 m^2^, *n* = 40). For renal-stage trend analyses and stage-specific analyses, G3a and G3b were combined as G3 (*n* = 105).

### 2.3 Samples collection and assay

Demographic and clinical data, including age, sex, body mass index (BMI), diabetes duration, and medication use, were collected from electronic medical records. After overnight fasting, venous blood samples were obtained in the morning for biochemical analyses. Serum uric acid (SUA), lipid profiles, albumin (ALB), HbA1c, and C-peptide levels were measured using an automated biochemical analyzer (Cobas 8,000, Roche Diagnostics, Mannheim, Germany).eGFR was calculated using the CKD-EPI equation. Continuous glucose monitoring (CGM) was performed using the Medtronic iPro2 system (Medtronic MiniMed, Northridge, CA, United States) for 72 h. CGM-derived metrics included time in range (TIR, 3.9–10.0 mmol/L), time above range (TAR), and time below range (TBR). The assays were performed according to the manufacturer’s quality-control procedures.

### 2.4 Statistical analysis

Statistical analysis was performed using SPSS 27.0 software. Continuous variables were expressed as mean ± standard deviation (SD) for normally distributed data or median (interquartile range) for non-normally distributed data. Categorical variables were expressed as numbers and percentages. Comparisons between two groups were performed using the independent-samples *t*-test or Mann Whitney U test, as appropriate. Comparisons among multiple renal stages were performed using one-way analysis of variance (ANOVA) or Kruskal Wallis test, as appropriate. Categorical variables were compared using the chi-square test.

Multivariable logistic regression analyses were performed to evaluate independent associations between nutrient metabolism indicators and DKD. Variables included in the models were selected based on clinical relevance and univariate analyses. Odds ratios (ORs) and 95% confidence intervals (CIs) were calculated. To explore renal-stage heterogeneity, additional stratified analyses were performed in G3 and G5 stages. A forest plot was constructed to visually summarize the stage-dependent associations of metabolic indicators. A two-sided *p* value <0.05 was considered statistically significant.

## 3 Results

### 3.1 Baseline characteristics of participants according to DKD status

Baseline characteristics of the study population are shown in Table 1. Compared with the NDKD group, patients with DKD were significantly older (*p* < 0.001) and had a longer duration of diabetes (*p* = 0.005). The proportions of lipid-lowering medication use (*p* = 0.004) and glucose-lowering medication use (*p* = 0.012) were significantly higher in the DKD group.

**Table 1:** Baseline characteristics of participants with and without DKD.

Variable	Non-DKD (*n* = 128)	DKD (*n* = 461)	Statistic	*p*
BMI	24.8 ± 3.1	25.2 ± 3.5	−1.21	0.226
Lipid-lowering	41 (32.0)	214 (46.4)	8.51	0.004
glucose-lowering medication	96 (75.0)	389 (84.4)	6.37	0.012
TIR, (%)	48 (30,63)	58 (41,76)	−4.238	<0.001
TAR≥10,%	42 (25,60)	41 (22,57)	−0.620	0.535
TAR≥13.9,%	10 (3,22)	11 (2,21)	−0.231	0.817
TBR ≤ 3.9,%	0 (0,1)	0 (0,1)	−0.669	0.504
TBR ≤ 3.0,%	0 (0,0)	0 (0,0)	−0.463	0.643
CGM, mmol/L	5.5 (4.5,6.9)	5.3 (4.3,6.7)	−0.731	0.465
Age, years	58 (47,66)	64.5 (53,72)	−3.691	<0.001
Sex, *n*(%)	/	/	10.571	0.001
Male	79 (61.7)	351 (76.1)	/	/
Female	49 (38.3)	110 (23.9)	/	/
Diabetes duration, years	10 (2,15)	10 (5,20)	−2.783	0.005
HbA1c,%	9.71 ± 2.35	9.81 ± 2.19	0.397	0.692
C-p, pmol/L	298.36 (176.36,555.15)	332.26 (209.69,553.93)	−0.572	0.568
C-p(2 h), pmol/L	740.44 (412.54,1272.74)	603.03 (353.81,1124.21)	−1.107	0.268

Data are presented as mean ± SD or median (Q1, Q3), as appropriate. Continuous variables were compared using the independent-samples *t*-test or Mann–Whitney U test, as appropriate. Categorical variables were compared using the chi-square test. “Statistic” represents the corresponding test statistic (t, Z, or χ^2^). DKD, diabetic kidney disease; NDKD, non-diabetic kidney disease; TIR, time in range; TAR, time above range; TBR, time below range; CGM, continuous glucose monitoring; HbA1c, hemoglobin A1c.

Among glucose metabolism indicators, TIR was significantly higher in the DKD group (*p* < 0.001). However, no significant differences were observed in TAR ≥10, TAR ≥13.9, TBR ≤ 3.9, TBR ≤ 3.0, CGM, HbA1c, fasting C-peptide, or 2-h C-peptide levels between the two groups (all *p* > 0.05). BMI and sex distribution were also comparable between groups.

Notably, patients with DKD demonstrated paradoxically higher TIR levels despite worse renal outcomes and greater treatment exposure. This finding suggests that the interpretation of glycemic metrics may become increasingly complex in patients with advanced renal dysfunction and intensive treatment settings.

Because several metabolic indicators differed between groups at the univariate level, multivariable logistic regression analysis was subsequently performed to identify metabolic indicators independently associated with DKD.

### 3.2 Multivariable logistic regression analysis of nutrient metabolism indicators associated with DKD

Multivariable logistic regression analysis was performed to identify independent metabolic indicators associated with DKD (Table 2). After adjustment for potential confounders, several variables remained significantly associated with DKD. Higher TIR was independently associated with lower odds of DKD. Specifically, for every 10% increase in TIR, the odds of DKD decreased by 3.0% (OR = 0.97, 95% CI: 0.95–0.99, *p* = 0.010). Age was also independently associated with DKD risk, with each additional year corresponding to a 5.8% increase in DKD odds (OR = 1.058, 95% CI: 1.012–1.105, *p* = 0.012). Higher serum uric acid remained an independent risk factor for DKD. For every 50 μmol/L increase in SUA, the odds of DKD increased by 21.0% (OR = 1.21, 95% CI: 1.08–1.31, *p* < 0.001). Similarly, each 1-unit increase in the TG/HDL-C ratio was associated with a 27.0% increase in DKD odds (OR = 1.27, 95% CI: 1.08–1.40, *p* = 0.002). In contrast, higher serum albumin was independently associated with lower DKD risk. Each 1 g/L increase in albumin was associated with a 9.0% reduction in DKD odds (OR = 0.91, 95% CI: 0.87–0.96, *p* < 0.001). Diabetes duration and BMI were not significantly associated with DKD after adjustment. These findings suggest that DKD is associated with multidimensional metabolic abnormalities involving glucose regulation, uric acid metabolism, lipid metabolism, and nutritional status.

**Table 2:** Multivariable logistic regression analysis of nutrient metabolic indicators associated with DKD.

Variable	OR	95% CI	*p*
TIR (per 10% increase)	0.97	0.95–0.99	0.01
Age	1.058	1.012–1.105	0.012
diabetes duration	1.018	0.972–1.067	0.455
BMI	1.18	0.79–1.77	0.425
SUA	1.21	1.08–1.31	<0.001
TG/HDL-C ratio	1.27	1.08–1.40	0.002
ALB	0.91	0.87–0.96	<0.001

Multivariable logistic regression analysis was performed with DKD as the dependent variable. The model was adjusted for age, sex, BMI, diabetes duration, and HbA1c, as applicable. TIR was entered per 10% increase; SUA per 50 μmol/L increase; TG/HDL-C ratio per 1-unit increase; and ALB per 1 g/L increase. OR, odds ratio; CI, confidence interval; TIR, time in range; SUA, serum uric acid; TG/HDL-C, triglyceride-to-high-density lipoprotein cholesterol ratio; ALB, albumin.

Although several metabolic indicators were independently associated with DKD in the overall cohort, DKD is a highly heterogeneous condition characterized by progressive renal dysfunction. Therefore, we further examined whether these metabolic patterns differed across renal stages.

### 3.3 Key metabolic indicators across renal stages with emphasis on G3 and G5

To provide a data-driven rationale for selecting G3 and G5 for stage-specific analyses, key metabolic indicators were compared across renal stages (Table 3). TIR showed a stepwise decline from G1 to G4, whereas SUA and TG/HDL-C ratio increased progressively with worsening renal function. Compared with G1 and G2, patients in G3 demonstrated more pronounced deterioration in glucose and lipid-related metabolic indicators, suggesting that G3 may represent a clinically important metabolic stage. In contrast, patients in G5 exhibited the lowest serum albumin levels among all renal stages, indicating substantial catabolic burden in end-stage renal dysfunction. These findings support the selection of G3 and G5 as two clinically distinct stages for subsequent stratified analyses.

**Table 3:** Comparison of key metabolic indicators across CKD stages in patients with T2DM.

Variables	G1 (*n* = 125)	G2 (*n* = 140)	G3 (*n* = 105)	G4 (*n* = 51)	G5 (*n* = 40)	P	*p*-trend
TIR, %, median (IQR)	61 (45–76)	58 (43–72)	52 (39–68)	49 (35–63)	50 (34–64)	0.018	0.009
SUA median (IQR)	328 (275–390)	348 (296–403)	412 (356–478)	468 (401–542)	521 (438–611)	<0.001	<0.001
TG/HDL-C median (IQR)	1.72 (1.18–2.35)	1.88 (1.29–2.49)	2.41 (1.74–3.21)	2.63 (1.88–3.47)	2.57 (1.79–3.39)	0.003	0.006
ALB, g/L, mean ± SD	42.8 ± 3.6	42.1 ± 3.5	40.9 ± 3.8	37.6 ± 4.2	33.8 ± 4.5	<0.001	<0.001

Data are presented as mean ± standard deviation (SD) for normally distributed variables or median (interquartile range) for non-normally distributed variables. Comparisons among renal stages were performed using the Kruskal–Wallis test. P for trend was calculated using linear regression analysis across worsening renal stages (G1–G5). TIR, time in range; SUA, serum uric acid; TG/HDL-C, triglyceride-to-high-density lipoprotein cholesterol ratio; ALB, albumin; eGFR, estimated glomerular filtration rate; KDIGO, Kidney Disease: Improving Global Outcomes.

In contrast, patients in G5 exhibited the lowest serum albumin levels among all renal stages, indicating substantial catabolic burden in end-stage renal dysfunction. These findings support the selection of G3 and G5 as two clinically distinct stages for subsequent stratified analyses, representing a metabolic transition window and a nutritional preservation stage, respectively.

### 3.4 Stage-specific metabolic analyses in G3 and G5 patients

Based on the progressive metabolic changes observed across G1–G5, G3 and G5 were selected as two clinically distinct stages for subsequent stratified analyses. To further explore stage heterogeneity of nutrient metabolism indicators in DKD, we performed stratified analyses in G3 and G5 renal stages (Table 4). In the G3 stage, higher TIR was independently associated with lower odds of DKD (OR = 0.85, 95% CI: 0.74–0.98, *p* = 0.026), while elevated SUA (OR = 1.19, 95% CI: 1.04–1.36, *p* = 0.012) and a higher TG/HDL-C ratio (OR = 1.26, 95% CI: 1.05–1.51, *p* = 0.014) were independently associated with higher odds of DKD. Serum albumin showed a borderline inverse association with DKD (OR = 0.93, 95% CI: 0.87–1.00, *p* = 0.052).

**Table 4:** Stage-specific multivariable logistic regression analysis of nutrient metabolic indicators associated with DKD in patients with CKD stages G3 and G5.

Variable	G3 OR (95% CI)	*p*	G5 OR (95% CI)	*p*
TIR	0.85 (0.74–0.98)	0.026	0.97 (0.85–1.11)	0.673
SUA	1.19 (1.04–1.36)	0.012	1.12 (0.99–1.27)	0.068
TG/HDL-C	1.26 (1.05–1.51)	0.014	1.10 (0.91–1.33)	0.325
ALB	0.93 (0.87–1.00)	0.052	0.85 (0.78–0.93)	0.001

Stage-specific multivariable logistic regression analyses were performed separately in G3 and G5 patients. Models were adjusted for age, sex, BMI, diabetes duration, and HbA1c, as applicable. TIR was entered per 10% increase; SUA per 50 μmol/L increase; TG/HDL-C ratio per 1-unit increase; and ALB per 1 g/L increase.

Table 4 supports stage-specific metabolic priorities in DKD: G3 appears to represent a window for glucose and lipid optimization, whereas G5 appears to be dominated by nutritional preservation and protein-energy status.

In contrast, in the G5 stage, lower serum albumin remained the only independent metabolic correlate of DKD (OR = 0.85, 95% CI: 0.78–0.93, *p* = 0.001), while the associations between TIR, SUA, and TG/HDL-C ratio and DKD were no longer statistically significant. These findings suggest that the dominant metabolic correlates of DKD may shift from glucose and lipid dysregulation in earlier renal impairment to protein-energy wasting in advanced renal dysfunction.

In G3 patients, TIR, SUA, and TG/HDL-C ratio remained significantly associated with DKD, indicating that glucose and lipid-related metabolic disturbances retain clinical relevance at this stage. In G5 patients, however, serum albumin emerged as the only significant metabolic correlate, while the associations of TIR and other metabolic indicators were no longer statistically significant. This pattern supports the view that nutritional depletion may become more prominent than glycemic discrimination in advanced renal dysfunction.

Taken together, these findings indicate that DKD in T2DM is characterized by a multidimensional metabolic profile rather than a purely glycemic abnormality. At the baseline level, DKD was associated with older age, longer disease duration, greater treatment exposure, and altered TIR profile. At the multivariable level, SUA, TG/HDL-C ratio, and serum albumin provided additional information beyond conventional glucose-related indices. Importantly, the relative importance of these metabolic correlates differed by renal stage, with G3 showing a profile dominated by glucose and lipid disturbances and G5 showing a profile dominated by nutritional depletion.

**Figure 1:**
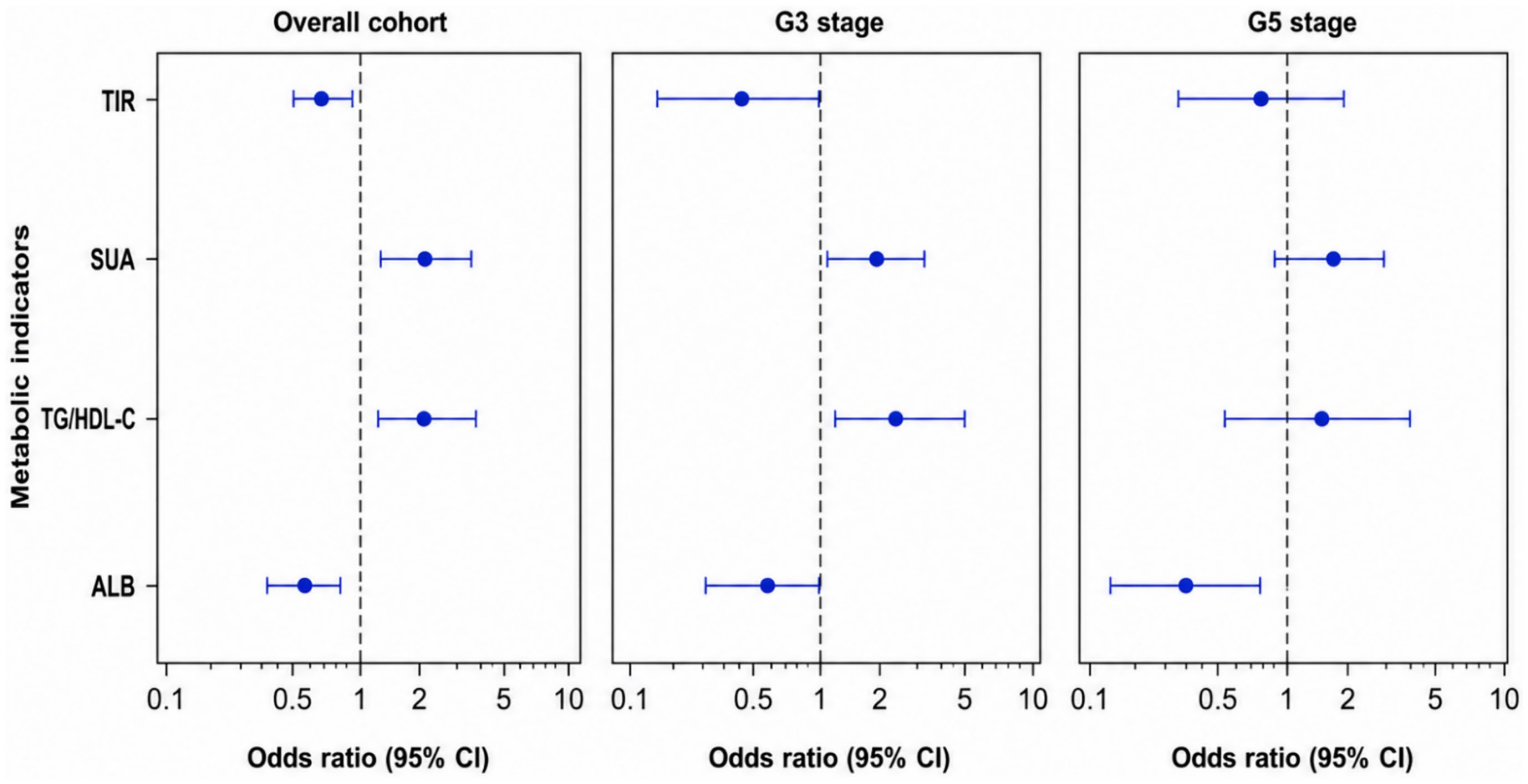
Forest plot of metabolic indicators across the overall cohort and renal stages. Odds ratios and 95% confidence intervals for TIR, SUA, TG/HDL-C ratio, and ALB are shown for the overall cohort, G3 stage, and G5 stage. The x-axis is presented on a logarithmic scale. The vertical dashed line indicates an odds ratio of 1.0.

To visually summarize the stage-dependent changes in metabolic correlates, a forest plot was constructed for the overall cohort, G3 stage, and G5 stage Figure 1. Forest plot of metabolic indicators across the overall cohort and renal stages. Odds ratios (ORs) and 95% confidence intervals (CIs) for TIR, serum uric acid (SUA), TG/HDL-C ratio, and albumin (ALB) are shown for the overall cohort, G3 stage, and G5 stage. The vertical dashed line indicates an OR of 1.0. Values to the left of the line indicate lower odds of DKD, whereas values to the right indicate higher odds of DKD.

## 4 Discussion

### 4.1 Principal findings

In this cross-sectional study of patients with T2DM, we investigated the association between DKD and multiple nutrient metabolism indicators, including CGM-derived TIR, serum uric acid, TG/HDL-C ratio, and serum albumin. This study provides several important insights into the multidimensional metabolic heterogeneity of DKD in patients with T2DM. First, patients with DKD showed older age, longer diabetes duration, greater use of glucose-lowering and lipid-lowering medications, and a paradoxically higher TIR compared with patients without DKD. Second, in the multivariable logistic regression model, higher TIR was modestly associated with lower odds of DKD, whereas higher SUA and TG/HDL-C ratio were associated with higher odds of DKD, and higher serum albumin was associated with lower odds of DKD. Third, renal-stage profiling showed progressive metabolic changes across G1–G5, including declining TIR, increasing SUA and TG/HDL-C ratio, and a marked reduction in albumin in G5. Finally, stage-specific analyses revealed distinct metabolic patterns between G3 and G5: in G3, TIR, SUA, and TG/HDL-C ratio remained significantly associated with DKD, whereas in G5, serum albumin was the only significant metabolic correlate.

Taken together, these findings suggest that DKD in T2DM is not merely related to glycemic status, but reflects multidimensional metabolic heterogeneity involving glucose metabolism, purine metabolism, lipid metabolism, and nutritional status. The principal novelty of this study lies not in demonstrating a general association between TIR and DKD, but in showing that the metabolic correlates of DKD may differ across renal stages, particularly between G3 and G5.

### 4.2 Interpretation of TIR in DKD

TIR is increasingly recognized as a useful CGM-derived metric that reflects the proportion of time spent within the target glucose range (26). Compared with HbA1c, TIR provides additional information regarding short-term glycemic exposure and daily glucose patterns (27). In our baseline comparison, patients with DKD had higher TIR than those without DKD. This finding appears counterintuitive because better glycemic control would generally be expected to correlate with lower complication risk. This paradoxical finding may reflect several factors. First, patients with DKD had a higher frequency of glucose-lowering medication use, suggesting more intensive glycemic management. Second, impaired renal function may reduce insulin clearance and alter glucose handling, thereby increasing the likelihood of lower glucose levels or hypoglycemia (28). Therefore, higher TIR in patients with established DKD should not necessarily be interpreted as superior long-term metabolic control.

However, this result should be interpreted cautiously. Patients with DKD had higher rates of glucose-lowering medication use, suggesting more intensive clinical management. In addition, renal dysfunction may alter glucose metabolism through reduced insulin clearance, decreased renal gluconeogenesis, appetite changes, and increased susceptibility to hypoglycemia (28). Therefore, higher TIR in patients with established DKD may reflect treatment intensity and altered renal physiology rather than truly lower lifetime glycemic burden. Future longitudinal studies should consider using time-varying covariates or propensity score methods to account for treatment intensity and to further clarify the causal relationship between glycemic management, TIR, and DKD outcomes.

After adjustment, TIR showed a modest inverse association with DKD in the overall model. This indicates that TIR remains clinically relevant, but its interpretation should not be separated from renal function stage and treatment context. This is consistent with the broader concept that CGM metrics may have different clinical implications in patients with preserved renal function and those with advanced kidney impairment.

### 4.3 Multidimensional nutrient metabolism and DKD

Beyond glucose metabolism, SUA, TG/HDL-C ratio, and serum albumin were independently associated with DKD in the overall model. This supports the concept that DKD is linked to multiple metabolic pathways rather than a single glycemic abnormality (29).

SUA reflects purine metabolism and renal urate handling. Elevated SUA may be associated with renal injury through oxidative stress, endothelial dysfunction, inflammation, and intrarenal vascular changes (30). In this study, higher SUA was associated with higher odds of DKD, suggesting that purine metabolism may provide additional information regarding DKD risk.

The TG/HDL-C ratio is a composite marker of atherogenic dyslipidemia and insulin resistance-related lipid disturbance (31). Its association with DKD suggests that lipid metabolism may be involved in diabetic renal injury. Compared with isolated lipid parameters, TG/HDL-C ratio may better reflect the combined effect of triglyceride elevation and HDL-C reduction, both of which are closely related to metabolic dysfunction in T2DM (32).

Serum albumin was inversely associated with DKD. However, albumin should not be interpreted solely as a nutritional marker (33). It may also reflect systemic inflammation, urinary protein loss, hydration status, hepatic synthesis, and overall catabolic burden. Therefore, the lower albumin level observed in advanced renal dysfunction may indicate a combined state of nutritional deterioration, inflammation, and protein loss rather than malnutrition alone.

### 4.4 Renal-stage metabolic evolution and rationale for selecting G3 and G5

A key feature of this study is the analysis of metabolic indicators across renal stages. Table 3 demonstrated that metabolic indicators changed progressively from G1 to G5. TIR showed a declining trend from G1 to G4, SUA and TG/HDL-C ratio increased with worsening renal function, and albumin showed a marked decline in G5. These findings suggest that the the metabolic profile of DKD appears to differ across renal stages, suggesting stage-specific metabolic heterogeneity rather than a fixed metabolic pattern.

G3 and G5 were selected for stage-specific analyses based on clinical and metabolic considerations. G3 corresponds to the clinically recognized threshold of moderate CKD, with eGFR falling below 60 mL/min/1.73 m^2^. At this stage, renal dysfunction is already established, but patients may still retain sufficient physiological reserve for metabolic management. Therefore, G3 may represent a clinically meaningful stage in which glucose stability, uric acid burden, and lipid metabolism remain relevant.

In contrast, G5 represents end-stage renal dysfunction. At this stage, patients are more likely to experience protein-energy wasting, inflammation, reduced appetite, altered glucose handling, and nutritional deterioration. In the present study, G5 showed the lowest albumin levels among all renal stages, supporting its interpretation as a stage with more prominent nutritional and catabolic burden.

Therefore, G3 and G5 were selected to represent two clinically meaningful and metabolically distinct renal stages rather than solely on the basis of statistical significance. G1 was not selected for stage-specific regression because it represents preserved renal function, where metabolic abnormalities are relatively mild and clinical intervention priorities are less distinct. G4 was also not selected as a primary focus because it represents an intermediate stage between metabolic transition and end-stage nutritional depletion, with less distinctive clinical interpretation. Therefore, G3 and G5 were chosen to represent two clinically meaningful and metabolically distinct states: a metabolic intervention window and a nutritional preservation stage. This selection was based on the disease trajectory and renal-stage metabolic profile, rather than solely on statistical significance.

### 4.5 Stage-specific metabolic transition from G3 to G5

The stage-specific regression analyses further supported the concept of stage-dependent metabolic evolution. In G3, higher TIR was associated with lower odds of DKD, while higher SUA and TG/HDL-C ratio were associated with higher odds of DKD. This suggests that, in moderate renal impairment, glucose stability, purine metabolism, and lipid metabolism remain clinically relevant.

In contrast, in G5, the associations of TIR, SUA, and TG/HDL-C ratio were no longer statistically significant, whereas serum albumin remained the only significant metabolic correlate. This suggests that in end-stage renal dysfunction, nutritional depletion may become more dominant than glycemic or lipid-purine metabolic indicators. This does not mean that glycemic control is unimportant in G5, but rather that its interpretation and clinical priority may differ from earlier renal stages.

This finding supports a stage-specific view of DKD progression. In earlier and moderate stages, metabolic control may remain central; in advanced stages, prevention of malnutrition and protein-energy wasting may become more clinically important. Collectively, these findings suggest that the metabolic phenotype of DKD evolves dynamically along renal function decline rather than remaining metabolically static throughout disease progression.

### 4.6 Implications for precision nutrition

The findings of this study may have implications for precision nutrition in DKD. Current nutritional management in DKD often stratifies protein intake according to renal function, but glycemic and lipid-related dietary strategies are still frequently applied in a relatively uniform manner. Our results suggest that nutritional priorities may need to change across renal stages.

For patients with preserved or moderately impaired renal function, particularly those in G3, nutritional strategies may focus on improving glycemic stability, reducing uric acid burden, and optimizing lipid metabolism. This may include individualized carbohydrate distribution, CGM-guided dietary adjustment, low-glycemic-index dietary patterns, purine management, and lipid-oriented dietary interventions.

For patients with advanced renal dysfunction, particularly G5, nutritional preservation may become the dominant priority. At this stage, excessive dietary restriction may increase the risk of malnutrition. Therefore, attention should be given to adequate energy intake, protein-energy status, prevention of hypoglycemia, monitoring of albumin, and assessment of inflammation and body composition.

These implications should be interpreted as hypothesis-generating. Because this study was cross-sectional and did not directly test dietary interventions, it cannot prove that stage-specific nutritional strategies improve renal outcomes. Nevertheless, the observed metabolic patterns provide a rationale for future prospective studies combining CGM, nutritional assessment, and renal-stage-specific dietary interventions. Importantly, the present study evaluated metabolic and nutrition-related biomarkers rather than direct dietary intake or nutritional interventions. Therefore, the proposed precision nutrition framework should be considered hypothesis-generating and requires validation in future prospective nutritional studies.

### 4.7 Strengths and limitations

This study has several strengths. First, it integrated glucose metabolism, purine metabolism, lipid metabolism, and nutritional status into a single DKD framework. Second, it examined metabolic indicators across renal stages, allowing a more nuanced interpretation of DKD heterogeneity. Third, by focusing on G3 and G5, this study highlighted two clinically distinct stages that may require different nutritional priorities.

Several limitations should also be acknowledged. First, the cross-sectional design prevents causal inference. Therefore, the observed associations cannot determine whether metabolic abnormalities contributed to DKD or resulted from renal dysfunction and treatment changes. Second, this was a single-center study, which may limit generalizability. Third, subgroup analyses in advanced renal stages may be limited by sample size, and the results should be validated in larger multicenter cohorts. Fourth, detailed dietary intake, protein intake, inflammatory markers, body composition, sarcopenia indices, and medication details were not fully evaluated. These factors may influence TIR, SUA, lipid metabolism, and albumin levels. Fifth, residual confounding cannot be excluded despite multivariable adjustment.

While direct measures of muscle mass (e.g., DEXA or bioelectrical impedance) were not available in this retrospective cohort, the profound and isolated drop in serum albumin observed in the G5 stage serves as a well-established surrogate marker for Protein-Energy Wasting (PEW)—a primary pathophysiological driver of sarcopenia in end-stage renal disease. These findings highlight the importance of integrating nutritional metabolism into DKD risk stratification and support the development of stage-specific precision nutrition strategies for patients with T2DM. In future clinical assessments of patients with G5 renal dysfunction, simple non-invasive sarcopenia screening tools, such as the SARC-F questionnaire and calf circumference measurement, may be incorporated together with ALB monitoring to improve risk stratification for nutrition-related non-classical complications.

## 5 Conclusion

In conclusion, DKD in T2DM is characterized by stage-specific nutrient metabolic heterogeneity involving glucose, lipid, purine, and protein-related metabolism. TIR remains an important glycemic indicator, but its interpretation appears to depend on renal stage and treatment context. G3 may represent a renal stage characterized by more prominent glucose, uric acid, and lipid metabolic abnormalities, whereas G5 may be characterized by more evident nutritional deterioration, with serum albumin showing the strongest association. These findings support an eGFR-informed precision nutrition framework for DKD. Because of the cross-sectional design, these findings should be interpreted as stage-specific associations rather than causal progression pathways. Future prospective studies are needed to validate these findings and determine whether renal-stage-specific nutritional strategies can improve clinical outcomes.

## Data Availability

The raw data supporting the conclusions of this article are not publicly available to protect participant confidentiality and privacy. All aggregated statistical data have been fully presented in the main text and tables. Additional methodological data can be provided upon reasonable request to XW, wangxiaohang1234@foxmail.com.
